# Case report: an unusual case of Brugada syndrome combined with a ventricular septal defect: A case report

**DOI:** 10.1097/MD.0000000000008695

**Published:** 2017-11-27

**Authors:** Xing Liu, Jianmei Zheng, Zhongcai Fan, Li Rao

**Affiliations:** a Department of Cardiovascular Medicine, West China Hospital, Sichuan University, Chengdu; b Department of Cardiovascular Medicine, The Affiliated Hospital of Southwest Medical University, Luzhou, Sichuan Province, China.

**Keywords:** Brugada syndrome, electrocardiogram, syncope, ventricular septal defect, whole exome sequencing

## Abstract

**Rationale:**

Brugada syndrome (BrS) is a cardiac ion channel disease that is caused by an autosomal dominant genetic abnormality. A ventricular septal defect is a common congenital heart disease, in which genetic defects play a significant role.

**Patient Concerns:**

We report an extremely rare case of a 42-year-old male with congenital heart disease, who suffered recurrent syncope and gastrointestinal bleeding. His electrocardiogram showed an unusual right bundle branch block-like pattern and ST-segment elevation in leads V1–V3.

**Diagnoses:**

The patient was eventually diagnosed with Brugada Syndrome Combined with a Ventricular Septal Defect.

**Interventions:**

The patient was treated with ICD implants.

**Outcomes:**

: We extracted his blood and performed whole exome sequencing. Whole exome sequencing revealed mutations in genes, which encode ion channels and proteins important for embryonic heart development. However, a novel mutation in the *SCN5A* gene was also found.

**Lessons:**

To our knowledge, this is the first genetically proven case of BrS combined with a ventricular septal defect.

## 1 Introduction

Brugada syndrome (BrS) is a genetic arrhythmogenic disease that has an estimated incidence between 4% and 12% in patients succumbing to sudden cardiac death (SCD)^[1]^ due to either ventricular tachycardia (VT) or ventricular fibrillation (VF).^[2]^ According to Antzelevitch et al, the incidence of BrS is higher in Southeast Asia and Japan, approaching 1%.^[3]^ Because SCD often occurs between 10:00 pm and 8:00 am of the next morning, it is called the “night of sudden death syndrome in Southeast Asia.” An electrocardiogram (ECG) is necessary to diagnose BrS. In 2005, a consensus report, published by the Second Consensus Conference of the European Society of Cardiology, proposed 3 types of abnormal ECG repolarization patterns (types 1–3) for BrS, of which only type 1 (characterized by ST-segment elevations in the right precordial leads) is diagnostic.^[4]^ However, the ACC/AHA/NASPE established new ECG criteria in 2012, declaring the existence of only 2 ECG patterns: pattern 1 (the coved pattern), identical to type 1 and pattern 2 (the saddle-back pattern) that includes ECG repolarization types 2 and 3.^[5]^ Nevertheless, the presence of either pattern advocates SCD prevention using an implantable cardioverter defibrillator (ICD).

More than 350 disease-causing gene mutations have been reported, including SCN5A, GPD1L, KCNE3, and CACNA1C.^[6]^

Ventricular septal defect (VSD) is the second most common congenital heart disease in Chinese adults. A large number of studies have shown that gene mutations play important roles in its etiology.^[7]^ We report an unusual patient who was diagnosed with BrS combined with a VSD; this patient subsequently underwent ICD implantation. The rapid shift of his ECG from an altered right bundle branch block (RBBB)-like pattern to a normal pattern suggested a special form of BrS. This patient also had a history of gastrointestinal hemorrhage. Therefore, we employed whole exome sequencing to determine whether this patient had unique mutations related to his disease state.

## 2 Case report

A 42-year-old male had suffered syncope 3 times in the 24 hours before his admission to the hospital. The first episode occurred at his home at 10:00 pm, resolving spontaneously within a few minutes. The second episode occurred 6 hours later, during deep sleep, followed by polypnea, sweating, and vomiting, but without accompanying limb tics; this episode also resolved within a few minutes. The last episode of syncope occurred at 7:00 pm at the hospital the next day. The patient had a history of an untreated VSD accompanied by gastrointestinal bleeding, which was previously controlled. There was no history of sudden death in any of his family members. His physical examination was unremarkable, except for a rough systolic heart murmur. All laboratory test results were normal, except for his stool, which was weakly positive for occult blood.

**Figure 1:**
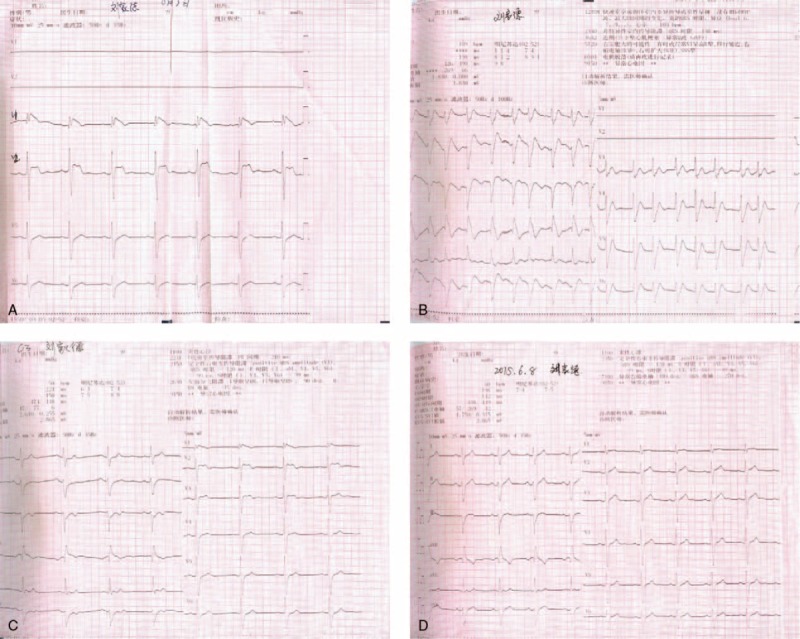
(A) The ECG right after syncope (taken on June 3, 2015). (B) The ECG of 15 min after the TdP showed the atrial fibrillation (AF) (taken on June 9, 2015). (C) The ECG of 2 d after syncope (taken on September 27, 2015). (D) The ECG of 6 d after syncope.

**Figure 2:**
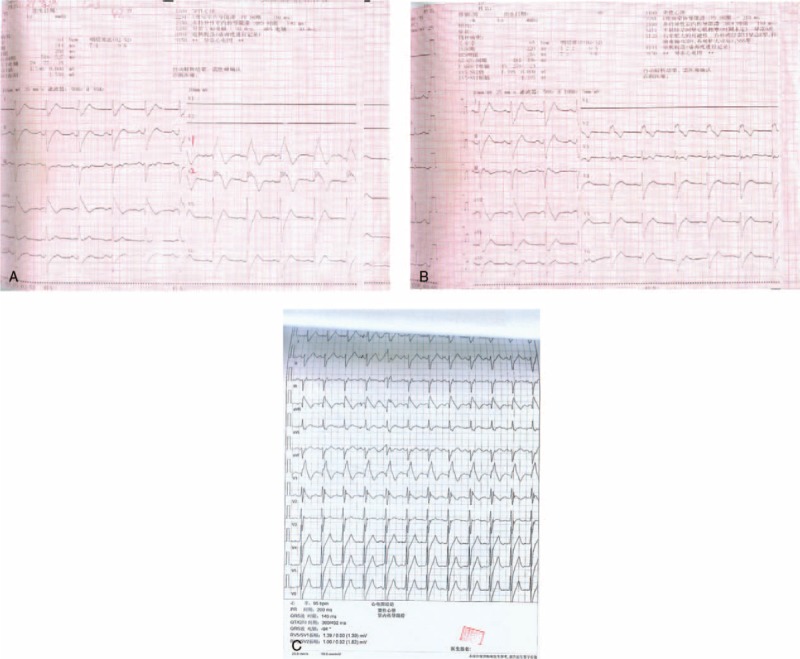
(A, B, C) A series of ECG which showed the odd RBBB-like pattern during the symptom-free periods.

**Figure 3:**
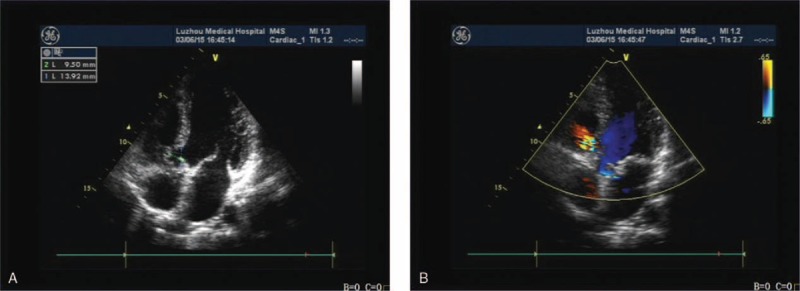
(A, B) Echocardiogram showing evidence of congenital heart disease with a perimembranous VSD. The largest diameter of the defect is 4.9 mm, with left to right shunting at the level of the ventricle.

On the day he fainted in the hospital, the ECG monitor revealed the torsades de pointes (TdP) form of VT. The VT was controlled by a defibrillator, and the ECG showed evidence of RBBB and ST-segment elevation in leads V1–V3, consistent with a classic type 1 BrS ECG pattern. This ECG pattern, which lasted 2 hours before returning to normal 2 days later, showed evidence of atrial fibrillation (AF) immediately after the TdP was controlled (Fig. 1A–D). Another ECG series showed an unusual RBBB-like pattern during the symptom-free periods (Fig. 2). Besides, the echocardiography confirmed the occurrence of VSD (determined to be a perimembranous type VSD) (Fig. 3).

According to his recurrent episode of syncope, VT, type I BrS ECG pattern, and echocardiography, a diagnosis of BrS combined with VSD was made. Considering his frequent syncope, he had a great risk of sudden death. So an ICD was implanted on June 8, 2015 to prevent SCD. The patient was treated orally with 2.5-mg bisoprolol (qid) to reduce ICD discharge. There were no recurrent episodes of syncope, and he was discharged 10 days later.

**Figure 4:**
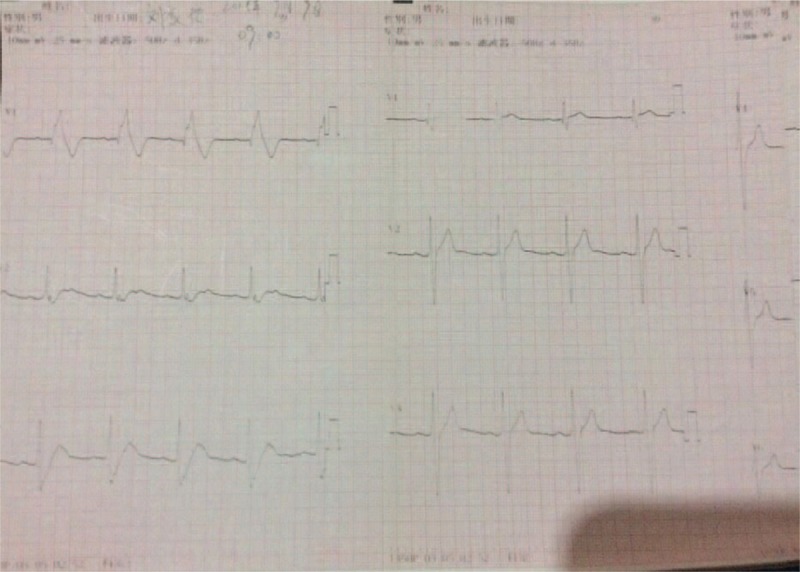
ECG rapidly shifting from an RBBB-like pattern to a normal pattern in leads V1–V3 within seconds, 1 mo after ICD implantation.

During the 3-month follow up, the patient experienced 2 shocks without any evidence of presyncope or syncope. However, at a previous follow-up, we recorded an ECG pattern that rapidly shifted from a RBBB-like pattern to a normal pattern in leads V1–V3 within seconds, and the patient had no symptoms at the time (Fig. 4).

**Figure 5:**
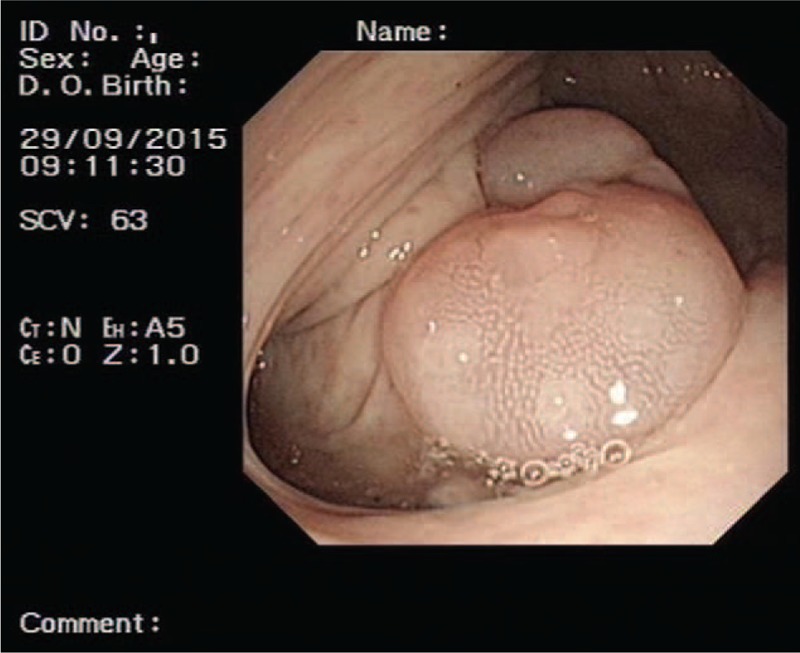
Result of colonoscopy: colonic polyp.

Three months later, the patient was seen by the gastroenterology department for gastrointestinal bleeding. Routine laboratory blood test indicated a hemoglobin level of 45 g/L. An enteroscopy was performed to determine the cause of the gastrointestinal bleeding (Fig. 5). During the procedure, the patient experienced sudden limb rigidity and lost consciousness, and recovering only after several seconds. VT was identified by ICD testing and was terminated by the ICD. After 1 month, the patient chose to discontinue Bisoprolol administration. Ten days later, he was discharged from the hospital, as the intestinal bleeding had resolved and the hemoglobin level had improved to 75 g/L. At the 10-month follow-up, ICD function was excellent, with no evidence of abnormal discharge, and the patient was well.

Both electrocardiography and echocardiography were done for the patient's sister and her sons, no specific findings were observed.

To obtain more information about this patient, we performed whole exome sequencing and found some novel mutations in genes related to ion channels and embryonic heart development. The generated sequencing data (12,222.55 Mb) resulted in a mean depth of 142.53 per base per targeted region, encompassing over 93.6% of the targeted genes. In total, we identified 42,038 single-nucleotide polymorphisms (SNPs) and 1559 insertion-deletion (indel) mutations from the exome of the proband (Appendices 1 and 2).

We then scored these SNPs and indels according to the filtering strategy as follows: quality score <20, depth ≤4, and synonymous, unknown, and non-frameshift mutations, resulting in 11,851 SNPs and 196 indels (displayed in forms 1 and 2). The next filter used a reserve frequency of ≤0.05, as predicted by 1000g2014oct_all, ESP6500, and Inhouse, resulting in only 4 SNPs and no indels (displayed in form 3). Lastly, 4 types of prediction software (SIFT, Poly Phen, Mutation Taster, and GERP) were used for analyses, identifying 3 SNPs, as shown in Table 1.

**Table 1:**
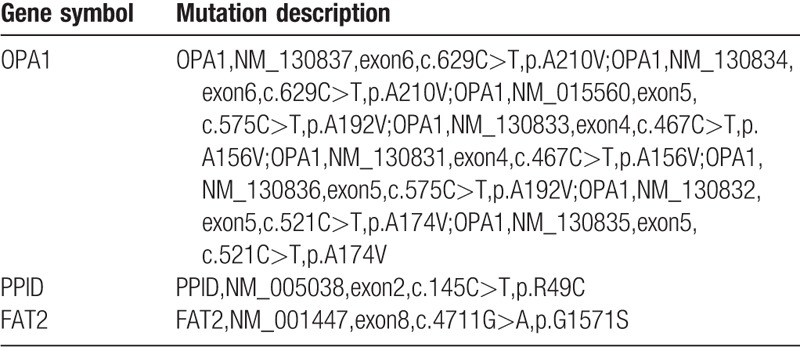
The predicted results by software: 3 SNPs in genes.

In addition, some positive mutations related to ion channel and the development of the embryonic heart were discovered in this patient, after >10,000 genetic mutations had been screened (Table 2).

**Table 2:**
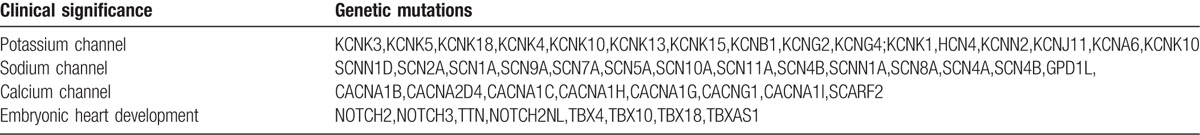
Table of genetic mutations found in this patient and their clinical significance.

## 3 Discussion

This case report describes a patient with a rare combination of BrS and VSD. There is no previous example of such a case in the medical literature. Both BrS and VSD are polygenetic or gene-related disorders. Whole exome sequencing was performed to assess whether these genetic diseases were linked or merely coincidental in this patient.

Nearly 20% to 25% of BrS patients possess a pathogenic variation in the *SCN5A* gene. The joint Canadian Cardiovascular Society/Canadian Heart Rhythm Society expert consensus report suggested that genetic testing for mutations of the *SCN5A* gene should be performed in patients diagnosed with BrS. There are 293 distinct BrS-associated mutations in SCN5A that have been linked to BrS symptoms.^[8]^ In this patient, the genetic variation in SCN5A was on exon 13: c.1960G > T, a stop-gain mutation. This mutation has not been previously reported.

In addition, mutations in other genes, such as *SCN9A*, *CACNG1*, *KCNG4*, *KCNJ12*, *KCNJ18*, and *KCNK15*, which are related to ion channels, were found in this patient by whole exome sequencing. A series of extraordinary RBBB-like ECG changes suggested a special form of BrS. However, more research is needed to determine whether the genetic mutations identified in this patient are associated with BrS or with these ECG changes directly. Mutations in these genes may lead to changes of stimulus threshold of the heart sodium channel, calcium channel, and potassium channel. And this patient had paroxysmal atrial fibrillation (PAF); this meant a greater chance of a malignant cardiovascular event. Kusano et al^[9]^ had confirmed that BrS patients are more likely to have AF, and that AF may predict a greater likelihood of VT and worse prognosis.

Variations in NOTCH2, NOTCH3, TTN, TBX4, TBX10, TBX18, and TBXAS1 are associated with congenital heart disease. The Notch signaling pathway plays a considerable role in heart development. Additionally, mutations in Notch receptors and their ligands have been identified as the causal agents of human congenital heart diseases, including VSDs.^[10]^ In this patient, similar genetic variations were found: NOTCH2, exon 2, c.137A > G, p.N46S; exon 2, c.112G > A, p.E38K; exon 1, c.57C > G, p.C19W; exon 2, c.137A > G, p.N46S; exon 2, c.112G > A, p.E38K; and exon 1, c.57C > G, p.C19W, which are all considered deleterious.

According to the sequencing results, many genetic variations were detected in this patient. Some of the SNPs and mutations are novel, and their functions are still not known. Further research is needed to determine the connection between the number of mutations and functional effects in similar patients. Additionally, more research is needed to determine if there is a relationship between BrS and VSD at a genetic level and whether certain genetic mutations result in unique clinical manifestations of BrS.

In addition, a solitary polyp rather than familial polyposis coli (FPC) is the cause of gastrointestinal bleeding in this patient. As FPC is a dominant autosomal dominant disease, its major causative gene is the *APC* gene, which is located at the long arm of chromosome 5, 5q21–22, and the mutation rate of APC in FPC is 30% to 50%. There is no evidence to prove that the APC is linked to heart disease. But, further studies are needed to investigate whether there is a relationship between genetic heart diseases and the presence of solitary polyp.

In summary, we present a patient with BrS, a VSD, an intestinal polyp, and an altered RBBB-like ECG, which suggest a rare etiology for ion channel disease. A novel mutation in SCN5A on exon 13–c.1960G > T, p.E654X–may be a new predictive marker for BrS or suggest a critical junction of multiple genetic maladies. Moreover, by comparing our whole exome sequencing results with those of other researchers, we can begin to understand, more completely, the influence of numerous genetic abnormalities on various disease states.
